# Markers of Sleep-Disordered Breathing and Prediabetes in US Adults

**DOI:** 10.1155/2012/902324

**Published:** 2012-07-05

**Authors:** Omayma Alshaarawy, Srinivas Teppala, Anoop Shankar

**Affiliations:** Department of Community Medicine, West Virginia University School of Medicine, 1 Medical Center Drive, P.O. Box 9190, Morgantown, WV 26506, USA

## Abstract

*Background*. Prediabetes is a preclinical stage in the hyperglycemia continuum where subjects are at increased risk of developing diabetes. Several studies reported a positive association between markers of sleep-disordered breathing (SDB) and diabetes. However, few studies investigated the relationship between SDB markers and prediabetes. *Methods*. We examined 5,685 participants ≥20 years from the National Health and Nutrition Examination Survey (NHANES) 2005–2008. The exposure of interest was SDB markers including sleep duration, snoring, snorting, and daytime sleepiness. The outcome was prediabetes (*n* = 2058), among subjects free of diabetes. *Results*. SDB markers were associated with prediabetes. Compared to those without any sleep disturbance, the multivariable odds ratio (OR) (95% confidence interval (CI)) of prediabetes among those with three or more SDB markers was 1.69 (1.28–2.22). In subgroup analyses, the association between SDB markers and prediabetes was stronger among women (OR (95% CI) = 2.09 (1.36–3.23) when compared to men (1.52 (1.00–2.35)) and was present among non-Hispanic whites (2.66 (1.92–3.69)) and Mexican Americans (1.99 (1.13–3.48)), but not among non-Hispanic blacks (1.10 (0.70–1.73)). *Conclusion*. SDB markers were associated with prediabetes. This association was stronger in women and was present mainly in non-Hispanic whites and Mexican Americans.

## 1. Introduction

Sleep-disordered breathing (SDB), a major public health problem, refers to a group of disorders characterized by abnormalities of respiratory pattern or the quantity of ventilation during sleep [1]. It has been estimated that approximately 12–18 million of US adults are affected by SDB [2]. On the other hand, prediabetes is a preclinical stage of diabetes, affecting more than 79 million Americans [3–5]. Newly identified prediabetes patients usually progress to type 2 diabetes in less than 3 years [6]. Emerging evidence suggests that lifestyle and pharmacologic interventions at the prediabetes stage can prevent or delay the progression into type 2 diabetes [7–9].

Several studies reported an association between SDB and an increased risk of diabetes [10–18] and fatal and nonfatal cardiovascular diseases [17, 19, 20]. However, there are only few studies investigating the relationship between markers of SDB and prediabetes [15, 16, 21, 22]. In this context, we examined the association between several commonly accepted markers of SDB including snoring, snorting, daytime sleepiness, and sleep duration and prediabetes in a nationally representative sample of US adults after accounting for age, sex, smoking, alcohol intake, BMI, serum lipids, and other potential confounders. We also examined the association between markers of SDB and prediabetes stratified by gender and race-ethnicity.

## 2. Methods

The data for this study is derived from the National Health and Nutrition Examination Survey 2005–2008. Detailed description of NHANES study design and methods are available elsewhere [23, 24]. The NHANES survey included a stratified multistage probability sample representative of the civilian noninstitutionalized US population. Selection was based on counties, blocks, households, and individuals within households and included oversampling of non-Hispanic blacks and Mexican Americans in order to provide stable estimates of these groups. Out of 20497 participants in NHANES survey 2005–2008, there were 10914 who were >20 years of age. We further excluded participants who were pregnant (*n* = 393), had prevalent cardiovascular disease (*n* = 1275), or have missing information on blood glucose levels, sleep variables, smoking status, depression, or other covariates included in the multivariable model. This resulted in 5685 participants included in the final analysis.

## 3. Main Outcome of Interest: Prediabetes

 Prediabetes was defined as a serum glucose ≥100 mg/dL and <126 after fasting for a minimum of 8 hours, or a 2-hour oral glucose tolerance test ≥140 and <200 mg/dL or glycosylated hemoglobin ≥39 (mmoL/moL) and <47.5 (mmoL/moL). Fasting plasma glucose levels were measured using hexokinase enzymatic method on Roche/Hitachi Modular P Chemistry Analyzer at the Fairview Medical Center Laboratory at the University of Minnesota, Minneapolis, Minnesota. Random plasma glucose levels were measured at the Collaborative Laboratory Services in Ottumwa, Iowa, using the Beckman Synchron LX20 in 2007 and the Beckman Coulter UniCel DxC800 in 2008. Glycosylated hemoglobin measurements were performed on the Tosoh 2.2 Analyzer (Tosoh Medics, Inc., 347 Oyster Pt. Blvd., Suite 201, So. San Francisco, CA 94080) in NHANES 2005-2006 and on the Automated HPLC System Glycohemoglobin Analyzer (Tosoh Medics, Inc., 347 Oyster Pt. Blvd., Suite 201, So.San Francisco, CA 94080) in NHANES 2007-08 at the University of Minnesota, Minneapolis Minnesota.

## 4. Assessment of Exposure

 Self-reported sleep habits were used to assess SDB, the exposure of interest. Examples of questions used are “How much sleep do you usually get at night on weekdays or workdays?”, “In the past 12 months, how often did you snore while you were sleeping?”, “In the past 12 months, how often did you snort, gasp, or stop breathing while you were asleep?”, and “In the past month, how often did you feel excessively or overly sleepy during the day?” Based on the responses to the previous questions, four sleep variables, sleep duration, snoring, snorting, and daytime sleepiness, were created. Sleep duration was categorized into ≤5, 6, 7, 8 and ≥9 h. Snoring and snorting variables were categorized into never or rare, occasional (3-4 nights/week), and frequent (5 or more nights/week). Daytime sleepiness was categorized into never or rare, sometimes (2–4 times/month), and often or almost always (5 or more times/month).

An additive summary SDB score combining all the four sleep variables was created by dichotomizing the individual variables based on their clinical significance and previous literature [18, 25, 26]. A score of 1 was assigned separately if the participants report sleep duration of ≤5, snoring at least 3-4 nights/week, snorting at least 3-4 nights/week, and daytime sleepiness at least 5 times/month. The summary score ranged from 0 to 4 corresponding to no sleep disturbance to coexistence of all 4 sleep disturbances.

## 5. Assessment of Covariates

Information on age, gender, race/ethnicity, smoking status, alcohol intake (g/day), level of education, and antihypertensive medication use was obtained during a standardized questionnaire at home interview. Educational attainment was categorized into less than high school graduate, high school graduate, and more than high school graduate. Individuals who had smoked <100 cigarettes during their lifetime were considered never smokers, those who had smoked ≥100 cigarettes lifetime and currently not smoking were considered former smokers, and those who had smoked ≥100 cigarettes lifetime and currently smoking were considered current smokers. Current alcohol drinking was defined as consumption of ≥1 alcoholic drink in the past 12 months. Moderate physical activity was defined as engaging in moderate-intensity sports, fitness, or recreational activities that cause a small increase in breathing or heart rate such as brisk walking, bicycling, swimming, or golf for at least 10 minutes continuously in a typical week. Information on anthropometric, physical, and laboratory components was obtained during the medical examination center (MEC) examination. Blood pressure (BP) was measured using a mercury sphygmomanometer and an average of three measurements was taken as the systolic and diastolic BP value. Patients were considered to have hypertension if they reported taking blood pressure reducing medication and/or had systolic BP ≥140 mm Hg or diastolic BP ≥90 mm Hg. Body mass index (BMI) was calculated as weight in kilograms divided by heights in meter squared. Waist circumference was measured in centimeters. Depression was defined as feeling down, depressed, or hopeless in the past 2 weeks for more than half of the days or nearly every day.

 Detailed description about the blood collection, processing, and quality control checks is provided in the Laboratory Procedures Manual [23, 27]. Serum C-reactive protein (CRP) was measured using latex-enhanced nephelometry. Total serum cholesterol was measured enzymatically using the Roche Hitachi 717 in 2005, Roche Hitachi 717 and 912 in 2006, and Roche Modular P chemistry analyzer in 2007-2008.

## 6. Statistical Analysis

We examined the association between categories of individual sleep variables, including snoring, snorting, daytime sleepiness, and sleep duration and prediabetes in two multivariable models. The first model was adjusted for age (years) and sex (female, male). The second model was additionally adjusted for race-ethnicity (non-Hispanic whites, non-Hispanic blacks, Mexican Americans, others), education (<high school, high school, >high school), smoking (never, former, current), current alcohol consumption (absent, present), moderate physical activity (times/week), body mass index (kg/m2), waist circumference (cm), depression (absent, present), systolic blood pressure (mmHg), C-reactive protein (mg/dL), total WBC count (mg/dL), and total cholesterol (mg/dL).

We created an additive summary SDB score and examined the association between this summary SDB score and prediabetes. Trends in the odds ratio of prediabetes across increasing summary SDB score were determined by modeling summary SDB score categories as an ordinal variable. We also performed subgroup analyses stratified by gender and race-ethnicity. All analyses were weighted to account for the unequal probabilities of selection, oversampling, and nonresponse using SUDAAN (version 8.0; Research Triangle Institute, Research Triangle Park, NC) and SAS (version 9.1.; SAS Institute, Cary, NC) software.

## 7. Results

Table 1 presents the baseline characteristics of the study population. Among 5685 participants included in the study, approximately 50% were women. The study population was primarily white (73.5%). Normal weight, overweight, and obesity were equally distributed. Mean C-reactive protein level was 0.36 (±0.01) mg/dL. Prediabetes was present in approximately 31% of the study subjects.

Table 2 presents the association between markers of SDB and prediabetes. We found that the SDB-prediabetes association varied among different markers. For sleep duration, participants who slept either ≤5 hours or 6 hours had higher odds of prediabetes when compared to those who slept 7 hours in the age, sex-adjusted model. The multivariable adjusted model showed a similar trend, although the odds ratios were attenuated and failed to reach statistical significance. For snoring, participants who occasionally or frequently snored had higher odds of prediabetes when compared to those who never or rarely snored in the age, sex adjusted, and the multivariable model. Similarly for snorting, participants who occasionally or frequently snorted had higher odds of prediabetes when compared to those who never or rarely snorted. The age, sex-adjusted odds ratios were statistically significant. The multivariable adjusted model showed similar trend, although it failed to reach statistical significance. For daytime sleepiness, participants who often and almost always sleep in the daytime did not have significantly higher odds of prediabetes in the age, sex-adjusted, or the multivariable-adjusted model.

 We also derived an additive summary SDB clustering score by dichotomizing the SDB variables and taking their sum, resulting in a score ranging from 0 (no or minimal markers of sleep disorder) to 4 (cooccurrence of all of the markers of sleep disorder). For the SDB clustering score, compared with a score of 0 (referent category), higher SDB clustering scores were associated with higher odds of prediabetes. The age, sex-adjusted model as well as the multivariable-adjusted model were statistically significant. 

Table 3 presents the association between prediabetes and the summary SDB clustering score by gender. The positive association between higher SDB clustering scores and prediabetes was still present, showing a stronger association among women when compared to men.

Table 4 presents the association between prediabetes and the summary SDB clustering score within various racial groups. The positive association between higher summary SDB clustering scores and prediabetes was consistently present among non-Hispanic Whites and Mexican Americans/others but was absent among non-Hispanic Blacks.

To further investigate the role of abdominal obesity, we ran a supplementary analysis where we additionally adjusted for waist circumference, in addition to BMI; our results were essentially similar. In men, compared to those with SDB summary score of 0 (referent), the multivariable odds ratio (95% CI) of prediabetes was 1.18 (0.89–1.57) for those with a score of 1, 1.22 (0.89–1.67) for those with a score of 2, and 1.53 (0.99–2.37) for those with a score of ≥3. Likewise, in women, compared to those with SDB summary score of 0 (referent), the odds ratio (95% CI) of prediabetes was 1.00 (0.82–1.21) for those with a score of 1, 1.05 (0.80–1.39) for those with a score of 2, and 1.97 (1.28–3.03) for those with a score of ≥3.

Also in another supplementary analysis where we additionally adjusted for total WBC count, another known biomarker for inflammation in addition to CRP, our results were analogous to our previous findings (Table 2). In the whole cohort, compared to those with a sleep summary score of 0 (referent), the multivariable odds ratio (95% CI) of prediabetes was 1.12 (0.94–1.33) for those with a score of 1, 1.16 (0.94–1.42) for those with a score of 2, and 1.66 (1.26–2.19) for those with a score of ≥3.

## 8. Discussion

 In a multiethnic sample of US adults who were free of diabetes, we found that markers of SDB including short sleep duration (≤6 h), occasional or frequent snoring, occasional or frequent snorting, and frequent daytime sleepiness were associated with prediabetes in initial age and sex-adjusted models. However, with multivariable adjustment for possible confounders, we found that only snoring and snorting were associated with prediabetes. In subsequent analyses, where we used an additive summary SDB clustering score as a measure of the cooccurrence of these SDB markers, we found that higher SDB clustering score was associated with higher odds of prediabetes, independent of age, gender, race-ethnicity, education, smoking, current alcohol consumption, moderate physical activity, body mass index, depression, systolic blood pressure, C-reactive protein, and total cholesterol. In subgroup analyses, the positive association between higher SDB summary scores and prediabetes showed a stronger association among women when compared to men and was consistently present among non-Hispanic whites and Mexican Americans but was absent among non-Hispanic blacks.

 Prediabetes is a preclinical stage in the hyperglycemia continuum where subjects are at increased risk of developing diabetes in the near future [5]. Although several previous studies reported a positive association between markers of SDB and diabetes mellitus [10–18], there are few studies investigating the relationship between markers of SDB and prediabetes. Among the few studies that have examined the association between SDB and prediabetes was the Western New York Study of 364 subjects where only short sleep duration was associated with prediabetes [22]. However, these results were inconsistent with other studies where both short and long sleep durations were positively associated with impaired glucose tolerance [15, 16, 21]. In the current study, where we studied a representative sample of US adults, we found that there was an overall positive association between markers of SDB and prediabetes, independent of confounders such as age, BMI, physical activity, and other factors.

 The clinical relevance of our findings is that (1) if these findings are replicated in future prospective studies, then interventions to treat SDB may be a novel strategy to prevent prediabetes, which is a recognized risk factor for diabetes [6, 7], as well as cardiovascular disease [28], and chronic kidney disease [29], and (2) from a risk stratification point of view, our results support the notion that subjects with clustering of ≥3 SDB symptoms should be screened for prediabetes.

In the current study, in subgroup analysis by gender, SDB was positively associated with prediabetes in both sexes. However, women showed a stronger association when compared to men. One explanation for this finding is that the association in men may be partially explained by factors that we adjusted in the multivariable model such as BMI, physical activity, and depression, whereas the association in women is relatively independent of these factors. However, it is also possible that there are true underlying gender differences in the association between SDB and prediabetes. Sleep apnea increases after menopause and can be reduced or alleviated with hormone replacement therapy [30–32]. In addition snoring was reported to increase during pregnancy [33, 34]. Similar gender differences were previously reported in studies investigating SDB and diabetes [11, 35]. However, to our knowledge; this is the first study analyzing the association between SDB and prediabetes by gender.

 Similarly, in subgroup analysis by race/ethnicity, the association between SDB and prediabetes was present among non-Hispanic whites and Mexican Americans/others, while the association was absent in non-Hispanic blacks. It is known that non-Hispanic blacks have a higher incidence of diabetes [36]. Moreover, non-Hispanic blacks are known to have higher prevalence of reduced sleep duration [37]. The underlying mechanism for the lack of association between SDB and prediabetes in non-Hispanic blacks remains unclear. Non-Hispanic blacks have higher BMI [38], higher rates of hypertension, and decreased physical activity [39], and it is possible that these traditional risk factors may explain most of the diabetes risk in non-Hispanic blacks than SDB. There is a need for more prospective studies to examine the association between markers of SDB and prediabetes by race/ethnicity to prove or disprove our findings.

 Several mechanisms have been proposed to explain the association between SDB and prediabetes. Elevations in evening cortisol concentrations and increased sympathetic activity as result of short sleep duration were found to predispose to insulin resistance [40]. The increase in the sympathetic activity due to sleep deprivation, daytime sleeping, and snoring is also associated with a decrease in glucose tolerance through an inhibitory effect on the pancreatic functions [41]. In addition, alterations in leptin secretion and elevated ghrelin in sleep-deprived subjects were reported to contribute to impaired glucose tolerance and insulin resistance through weight gain [13, 42–44]. However, in the current study, the association between short sleep duration and prediabetes remained significant even after adjustment for BMI and waist circumference suggesting that there probably are mechanisms other than weight gain present in the association between short sleep duration and prediabetes. Furthermore, experimental studies have shown that reduced sleep is associated with increased levels of inflammatory markers such as tumor necrosis factor alpha, interleukin 6, and C-reactive protein [20, 45]. Inflammation has in turn been shown to predict type 2 diabetes [46, 47]. Snoring and associated sleep apnea may play a role in the development of insulin resistance through increased catecholamine and cortisol levels [48–50]. For daytime sleeping, the increased sympathetic activity on awakening from naps results in activation of the renin-angiotensin system, which is suggested to modulate insulin resistance and associated hyperglycemia [51, 52].

 This study has numerous strengths. Ours is one of the few studies investigating the relationship between markers of SDB and prediabetes. In addition, to our knowledge, this is the first study analyzing the association between SDB and prediabetes by gender and race/ethnicity. Moreover, the large national sample of racially and ethnically diverse subjects and the ability to adjust for numerous potential confounders add to the strengths of the study. However, the study has some limitations worth mentioning. First, the cross-sectional nature of the study limits making conclusion regarding the temporal association between SDB and prediabetes. In addition, markers of SDB were self-reported and this might have resulted in a misclassification bias.

 In conclusion, in a nationally representative sample of adults who were free of diabetes, we found that markers of SDB were associated with prediabetes independent of confounders, such as age, gender, BMI, and other factors. This association was stronger in women and present mainly in non-Hispanic whites and Mexican Americans.

## Figures and Tables

**Table 1:** Baseline characteristics of the study population*.

Characteristics	Whole cohort (*n* = 5685)
Women (%)	2782 (50.6)
Age (years)	43.7 ± 0.4
Race/ethnicity (%)	
Non-Hispanic whites	2898 (73.5)
Non-Hispanic blacks	1085 (9.4)
Mexican Americans	1021 (7.8)
Others	681 (9.3)
Education categories (%)	
Below high school	1409 (16.1)
High school	1391 (24.3)
Above high school	2885 (59.5)
Smoking (%)	
Never smoker	3045 (52.9)
Former smoker	1284 (22.9)
Current smoker	1356 (24.2)
Current drinker (%)	4056 (76.3)
Moderate physical activity (%)	2741 (54.9)
Body mass index (%)	
Normal weight	1869 (35.2)
Overweight	2036 (34.6)
Obese	1780 (30.1)
Depression (%)	348 (5.0)
Systolic blood pressure (mm Hg)	119.7 ± 0.3
C-reactive protein (mg/dL)	0.358 ± 0.011
Total cholesterol (mg/dL)	200.74 ± 0.78
Prediabetes (%)	2058 (31.3)

*Data presented are number (percentages) or mean values ± standard error (SE), as appropriate for the variable.

**Table 2:** Association between sleep variables and prediabetes.

Sleep variables	Number at risk (% prediabetes)	Age, sex adjusted odds ratio (95% CI)	Multivariable-adjusted odds ratio* (95% CI)
Sleep duration (hours)			
≤5 hrs	840 (35.2)	1.40 (1.15–1.71)	1.19 (0.97–1.46)
6 hrs	1332 (34.0)	1.23 (1.03–1.47)	1.17 (0.96–1.42)
7 hrs	1676 (30.0)	1 (referent)	1 (referent)
8 hrs	1485 (29.0)	0.95 (0.78–1.15)	0.94 (0.77–1.14)
≥9 hrs	352 (30.8)	1.13 (0.71–1.81)	1.06 (0.64–1.74)

*P*-trend		0.005	0.07

Frequency of snoring			
Never or rarely	2860 (25.8)	1 (referent)	1 (referent)
Occasionally	1072 (33.8)	1.39 (1.17–1.64)	1.24 (1.04–1.47)
Frequently	1753 (38.5)	1.55 (1.33–1.82)	1.21 (1.02–1.43)

*P*-trend		<0.0001	0.02

Frequency of snorting			
Never or rarely	5034 (29.9)	1 (referent)	1 (referent)
Occasionally	341 (41.5)	1.42 (1.05–1.92)	1.24 (0.92–1.68)
Frequently	310 (42.4)	1.51 (1.12–2.04)	1.22 (0.91–1.63)

*P*-trend		0.001	0.07

Frequency of daytime sleepiness			
Never or rarely	3287 (32.0)	1 (referent)	1 (referent)
Sometimes	1482 (30.3)	1.01 (0.84–1.22)	1.00 (0.82–1.21)
Often and almost always	916 (30.9)	1.17 (0.98–1.39)	1.11 (0.92–1.33)

*P*-trend		0.14	0.37

Sleep summary score			
0	2146 (25.8)	1 (referent)	1 (referent)
1	2228 (33.1)	1.34 (1.14–1.57)	1.14 (0.96–1.35)
2	974 (35.4)	1.46 (1.21–1.76)	1.18 (0.97–1.43)
≥3	337 (44.2)	2.32 (1.78–3.04)	1.69 (1.28–2.22)

*P*-trend		<0.0001	0.0007

*Adjusted for age (years), sex (men, women), race-ethnicity (non-Hispanic whites, non-Hispanic blacks, Mexican Americans, others), education (<high school, high school, >high school), smoking (never, former, current), current alcohol consumption (absent, present), moderate physical activity (times/week), BMI (kg/m^2^), depression (absent, present), systolic BP (mm Hg), CRP (mg/dL), and total cholesterol (mg/dL).

**Table 3:** Association between sleep variables and diabetes mellitus, by gender.

Sleep summary score	Number at risk (% prediabetes)	Age adjusted odds ratio (95% CI)	Multivariable-adjusted odds ratio* (95% CI)
Men			

0	956 (30.2)	1 (referent)	1 (referent)
1	1180 (36.6)	1.26 (0.99–1.69)	1.18 (0.89–1.57)
2	569 (39.1)	1.43 (1.06–1.93)	1.22 (0.90–1.66)
≥3	198 (44.4)	1.94 (1.29–2.91)	1.52 (1.00–2.35)

*P*-trend		0.0005	0.05

Women			

0	1190 (22.7)	1 (referent)	1 (referent)
1	1048 (29.3)	1.39 (1.15–1.67)	1.05 (0.86–1.29)
2	405 (30.4)	1.50 (1.19–1.89)	1.11 (0.85–1.46)
≥3	139 (44.0)	3.02 (2.00–4.58)	2.09 (1.36–3.23)

*P*-trend		<0.0001	0.009

*Adjusted for age (years), race-ethnicity (non-Hispanic whites, non-Hispanic blacks, Mexican Americans, others), education (<high school, high school, >high school), smoking (never, former, current), current alcohol consumption (absent, present), moderate physical activity (times/week), BMI (kg/m^2^), depression (absent, present), systolic BP (mm Hg), CRP (mg/dL), and total cholesterol (mg/dL).

**Table 4:** Association between sleep variables and diabetes mellitus, by race/ethnicity.

Sleep summary score	Number at risk (% prediabetes)	Age, sex adjusted odds ratio (95% CI)	Multivariable-adjusted odds ratio* (95% CI)
Non-Hispanic whites			
0	1125 (24.7)	1 (referent)	1 (referent)
1	1111 (31.5)	1.36 (1.11–1.68)	1.19 (0.95–1.49)
2	492 (34.6)	1.56 (1.24–1.97)	1.28 (1.00–1.64)
≥3	170 (43.3)	2.66 (1.92–3.69)	1.92 (1.35–2.72)

*P*-trend		<0.0001	0.0005

Non-Hispanic blacks			

0	391 (35.7)	1 (referent)	1 (referent)
1	399 (37.6)	0.95 (0.70–1.30)	0.80 (0.57–1.11)
2	210 (42.0)	1.17 (0.81–1.70)	0.93 (0.62–1.38)
≥3	85 (41.5)	1.10 (0.70–1.73)	0.80 (0.50–1.23)

* P*-trend		0.39	0.41

Mexican-Americans/others			

0	630 (25.9)	1 (referent)	1 (referent)
1	718 (37.2)	1.43 (1.16–1.76)	1.13 (0.91–1.41)
2	272 (34.7)	1.15 (0.78–1.69)	0.90 (0.61–1.32)
≥3	82 (51.1)	1.99 (1.13–3.48)	1.52 (0.85–2.72)

*P*-trend		0.02	0.49

*Adjusted for age (years), sex (men, women), education (<high school, high school, >high school), smoking (never, former, current), current alcohol consumption (absent, present), moderate physical activity (times/week), BMI (kg/m^2^), depression (absent, present), systolic BP (mm Hg), CRP (mg/dL), and total cholesterol (mg/dL).
